# Commentary: Added sugar intake and its associations with incidence of seven different cardiovascular diseases in 69,705 Swedish men and women

**DOI:** 10.3389/fpubh.2025.1592684

**Published:** 2025-08-11

**Authors:** Andrzej Porębski

**Affiliations:** Faculty of Law and Administration, Jagiellonian University, Kraków, Poland

**Keywords:** reverse causality, confounding, causal inference, underreporting, added sugar, dietary sugars, cardiovascular disease, alcohol consumption

## 1 Introduction

The paper (1) provides strongly unexpected results. First, the group consuming the least added sugar had the highest risk of cardiovascular disease (CVD) in the study. Second, groups consuming more sugary treats (even >14/week) had a lower risk of CVD than the group consuming the least. These results aroused my curiosity. After taking a closer look, I concluded that the study is probably fraught with the problem of reverse causality. This problem appears to provide the most plausible explanation for the bizarre results, which, misinterpreted, would suggest that it would be better to eat sweets, and certainly not eliminate them from the diet. In this commentary, which relies on observational critique, I consecutively consider the two signalled results in light of the problem of reverse causality. I argue that a low intake of added sugar or sweet treats does not carry an increased risk of CVD, and that such a result is a correlation that emerged due to a flawed study design.

## 2 The reverse causality problem and the added sugar intake

In a strict sense, reverse causality occurs when the outcome (dependent variable) causes the exposure (predictor, independent variable); in a broad sense, reverse causality is also confounding—that is, a situation where a confounder closely related to the outcome (e.g., a disease when mortality risk is investigated) impacts simultaneously on the outcome and the exposure (2). No matter how exactly we define it, reverse causality burdens many studies, especially those in dietetics or epidemiology (2–6), and the extent of this problem seems underexamined (7). I believe that reverse causality severely burdens this study (1), making the harm of added sugar and sugary treats arguably underestimated.

The paper states, “for a majority of the outcomes, however, the highest risks were found in the lowest intake category”—that is, in the group in which 5% or less of the non-alcoholic energy intake comes from added sugar (the ≤ 5E% group). Figure 2 and Table 3 in the commented study (1) present mainly U-shaped and L-shaped curves. However, such curves—in the absence of a known biological mechanism explaining them or even with a known mechanism opposite to that suggested by the study—may occur due to reverse causality (4, 7–9). The group with the lowest added sugar intake (approximately 21% of the sample) is undoubtedly specific—almost complete elimination of added sugar is not typical behaviour. The significantly increased health risks observed in this group seem a more plausible explanation for such a decision relative to the reverse, in which the non-consumption of added sugar would worsen cardiovascular health. Thus, the proposed mechanism of reverse causality is as follows: as one's health deteriorates, one is more likely to eliminate products one considers unhealthy from the diet. This could be a personal decision or compliance with the recommendation of a physician or a nutritionist. Thus, it was not the low sugar intake that caused the disease (the outcome) but, on the contrary, the disease (or the risk of disease) was the reason for the decision to reduce sugar intake.

**Figure 1:**
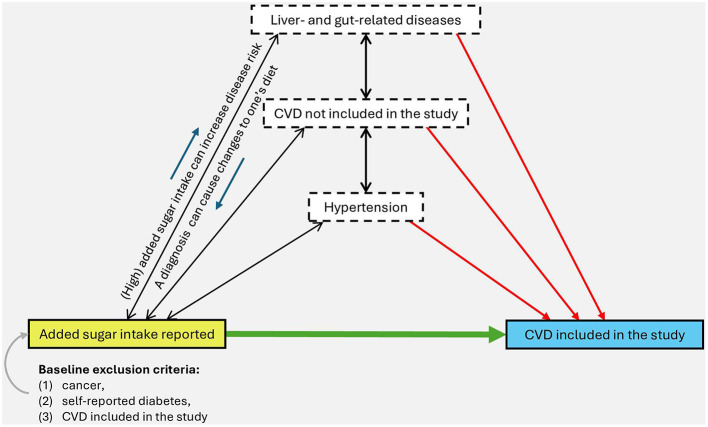
Possible causal links between the exposure (yellow rectangle), the outcome (blue rectangle), and some of the variables uncontrolled in the study (white rectangles). Arrows and their directions represent the direction of *possible* causal relationships—the presence of the arrows does not imply the certainty of the occurrence of such relationships but the possibility of their occurrence, which would have to be properly scrutinized to precisely determine the nature of the exposure relationship with the outcome. This is an illustrative figure only and cannot be treated as a complete causal analysis. Source: own elaboration.

Regarding the aforementioned mechanism of reverse causality, it should be noted that the study used baseline exclusions of the observations for patients having (1) cancer, (2) self-reported diabetes, or (3) at least one of eight selected CVDs, specifically: ischemic stroke, intracerebral haemorrhage, subarachnoid haemorrhage, myocardial infarction, heart failure, atrial fibrillation, aortic valve stenosis, and abdominal aortic aneurysm [see the label of Figure 1 in the commented study (1)]. Unfortunately, the list of CVDs resulting in baseline exclusion included only a subset of all CVDs. Thus, there were no exclusions due to, for example, peripheral arterial disease or coronary artery disease (both are common CVDs). Therefore, included in the sample of the study may have been people with common CVDs at the outset, other than those considered in the study.

Moreover, there are various other diseases that cause an increase in CVD risk or a general deterioration in health but that are strong motivators for improving one's diet. In particular, liver- and gut-related diseases can both negatively impact sugar metabolism (hence, can prompt the decision to reduce added sugar in one's diet) and increase CVD risk (10, 11), and, thus, people having these conditions should be excluded from the sample. Finally, if a disease (including cancer) appeared in a later part of the study, before the second questionnaire was completed, it was no longer controlled, unless it was a specific CVD considered in the study. Therefore, it is difficult to see the baseline exclusion mechanism used in the study as preventing the possibility of reverse causality occurring in the manner indicated.

Another mechanism potentially explaining the study results is the well-documented phenomenon of the selective underreporting of intake (12, 13). It is possible that the ≤ 5E% group did not consume the least amount of added sugar, but rather, due to health problems or medical recommendations, tended to underreport their added sugar intake (as something embarrassing, indicating non-compliance with recommendations). Then, the higher health risks would translate into a higher probability of underreporting and, consequently, a lower observed (though not actual) added sugar intake.

Moreover, the study did not control for factors such as hypertension (or, more generally, blood pressure), glycaemia, sodium intake, lipid levels, family CVD history, and economic status. I do not state that controlling all these factors is easy, and I know that relevant data is not always available. Unfortunately, the omission to control too many potentially important factors renders the problem of (broadly understood) reverse causality even more plausible. For instance, hypertension is a frequent cause of CVD (the outcome of the study) and, at the same time, the diagnosis of hypertension can cause significant changes to one's diet, for example, a reduction in added sugar consumption (the exposure). In the absence of these controls, it is difficult to say whether the respondents with the lowest sugar intake are not simply diseased people. Figure 1 presents an example of the uncontrolled variables in the study and their possible influence on the exposure and outcome, which makes it impossible to infer the relationship between low added sugar intake and higher CVD risk.

Contrary to the authors' suggestions, the risk of reverse causality was not sufficiently reduced by the sensitivity analysis they conducted, since the analysis consisted only of removing some observations in which certain CVDs manifested themselves most rapidly—during the first 3 years of follow-up. Such a procedure may indeed have reduced the problem of reverse causality, but only to the extent that it was due to the presence of the initial stage of the (undiagnosed) disease just before the follow-up stage. Meanwhile, other and even more important potential drivers of reverse causality—such as a baseline exclusion based only on some forms of CVD and a failure to control for, for example, hypertension—were also present in the sensitivity analyses.

## 3 Sugary treats as an antidote for cardiovascular disease?

**Figure 2:**
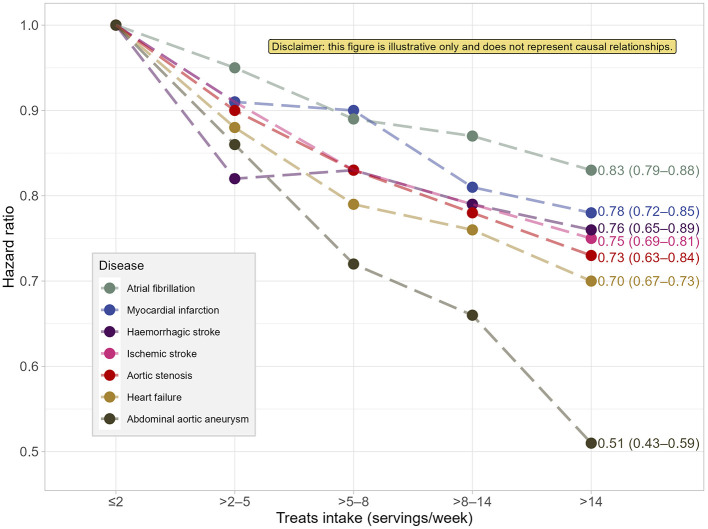
Estimated hazard ratios of selected cardiovascular diseases risk in relation to weekly intake of sugary treats reported in the study (1) (based on the multivariate standard method of energy adjustment). Labels indicate hazard ratios for the >14 treats/week group, with their 95% confidence intervals in parentheses. This figure is a part of an argument against the soundness of the presented results and, consequently, the author of the commentary recommends not to treat it as a separate and meaningful outcome. Source: own elaboration based on the data from Supplementary Table S16 in Janzi et al. (1). This figure was created using *R* language, version 4.4.0, including packages: *tidyverse* (14), *ggrepel* (15), and *ggpp* (16).

I focused earlier on the strangely increased risk reported for the group consuming the least added sugar. However, the most compelling argument for the reverse causality phenomenon lies in the commented study supplement, according to which, regarding the risk of CVD, it would be healthier to eat >14 sugary treats per week than to eat ≤ 2 (*sic!!!*), see Supplementary Tables S3–S9, S16 in (1). In Table S16, one can even see that this is a systematic tendency: All coefficients (hazard ratios) except those for the ≤ 2 treats/week group (the reference and the lowest intake group) are less than 1, and most of them are statistically significant (see Figure 2 of this commentary). In the most extreme case of abdominal aortic aneurysm as an outcome, the ratio for the >14 treats/week group vs. the ≤ 2 group is 0.5, which would suggest some kind of magical shielding effect of consuming sweet treats, although the model for added sugar in the same study shows a trend of increased risk for higher sugar consumption (Supplementary Table S9, Main model). This internal inconsistency of the results for abdominal aortic aneurysm—between the models for frequency of treats consumption and the others—provides another rationale for reverse causality. The most plausible explanation of the effect is the reverse causality and the systematic underreporting of sugar treats (i.e., foods with a negative health image) (12).

## 4 Discussion

Three issues are worth discussing further. First, I would like to draw an analogy between the problems described and the problems of studying the effects of alcohol on health. The issue of reverse causality led many previous studies on the relationship between alcohol and health to suggest that low alcohol consumption was significantly healthier than no alcohol consumption. The main reason for this was that some in the non-drinking group were alcoholics or did not drink alcohol due to poor health. Recent large-scale studies such as (4, 8, 9) have used Mendelian randomisation (more robust to reverse causality), or adequately controlled for factors correlated with (not) drinking alcohol or eliminated non-drinkers for health reasons from the sample. These methodological advancements mitigated the problem of reverse causality—as well as measurement problems with reporting categories such as abstinence (17), analogous to intake underreporting. Consequently, it has been determined that the “protective value of small amounts of alcohol” is either non-existent or much smaller than originally assumed. I am concerned that the research community has not assimilated the lessons from the studies leading to erroneous conclusions on the effects of alcohol on health.

Second, the authors' main comment in the discussion of the aforementioned inconsistencies and bizarre findings on sweet treats is that they are consistent with two other studies (18, 19). However, both of these studies were produced by a similar team, have similar results, and are probably burdened with similar problems. To put it directly, a similar commentary could be written for each of them. This is a worrisome trend: getting consistent results according to which binge-eating sweets is associated with reduced health risks should suggest the need to revise the methodology rather than doing more studies that “confirm” earlier results. The authors' vigilance should have been previously aroused by studies providing competing evidence, such as (20). Specifically, study (20) features relatively good confounders control and shows an exponentially increasing risk for CVD mortality as the percentage of energy intake from added sugar increases.

Third, the commented study deals with an important and extremely media-savvy topic, as evidenced by the high reach the study has achieved (a >1,000 Altmetric score in < 5 days). However, such an extensive reach of some topics comes with a special responsibility. The commented paper could easily be misinterpreted as an indication that it is better to eat added sugar than not to eat it. For example, I read about the study just a day after its publication in a Polish-language high-reach newspaper containing a separate section on the popularisation of science (21). The translated title of this article is “Too much sugar? Unhealthy. Too little? Also not good! A new study” (21). It is easy to see that the study results are interpreted without considering the issue of reverse causality. Similarly, they can be misinterpreted in policy-making and even in legislation. Even more so, the issue of reverse causality should be transparently considered and discussed by researchers. This problem should be prevented, whether by Mendelian randomisation, by careful control of confounders and thoughtful adjustment of the sample by appropriate baseline exclusions, by objectifying measurements and shying away from out-of-control self-reporting, or by causal inference tools such as directed acyclic graphs. If, on the other hand, the problem of reverse causality may burden the study, the results should be discussed with an indication that they may represent some kind of “correlational artefact”.
